# Association between disability and cardiovascular event and mortality: A nationwide representative longitudinal study in Korea

**DOI:** 10.1371/journal.pone.0236665

**Published:** 2020-07-30

**Authors:** Ki Young Son, Seung Hee Kim, Sung Sunwoo, Ji-Yun Lee, Seongmi Lim, Young Sik Kim

**Affiliations:** 1 Department of Family Medicine, Asan Medical Center, University of Ulsan College of Medicine, Seoul, Republic of Korea; 2 College of Nursing, Chung-Ang University, Seoul, Republic of Korea; Nagoya University, JAPAN

## Abstract

This study aimed to examine the association between disability and cardiovascular (CV) disease incidence and mortality in Korea longitudinally, using a national representative sample. We used the National Health Insurance Service-National Health Screening Cohort (NHIS-HEALS) database, which includes information on the disability of the National Screening Program participants such as severity and type of disability, which were obtained from the Korean National Disability Registry. Cox proportional hazard models were used to evaluate the association between disability and CV disease incidence and mortality. We constructed four models with different levels of adjustment, in which Model 3 was a fully adjusted model. This study included 514,679 participants, and 7,317 CV deaths were reported within a mean follow up of 10.8 ± 3.9 years (maximum, 13.9 years). For 5,572,130 person-year (PY) follow-up, the CV mortality rate was 1.313 per 1,000 PY. In Models 1 and 2, CV disease incidence was significantly higher in participants with disability than in those without disability. In Model 3, the incidence was higher only among participants aged 50–64 years and severe disabled participants aged <50 years. CV mortality was significantly higher in participants with disability than in those without disability in all Models, and the mortality increased in both sexes in Models 1 and 2 but only increased in men in Model 3. Similar results were observed in the subgroup analysis of health behavior and chronic diseases. People with disability showed higher CV disease incidence and mortality than those without disability, regardless of the type of disability or risk factors for CV disease.

## Introduction

Disability is defined by the International Classification of Functioning, Disability and Health as the state of having limitations in body functions, structures, activities, and participation [1]. According to the National Disability Registry (NDR) of Korea, over 2.4 million individuals were registered as people with disability, which accounted for 5% of the Korean population [2].

Despite the importance of health in people with disabilities, they have received little attention within the health care system [3, 4]. A previous study in Korea showed that people with disability were more likely to be physically inactive, have higher incidence of osteoporosis, to be underweight, have suicidal thoughts, and have impaired quality of life [5]. Several studies in western countries also showed that people with disability often have adverse health behaviors (i.e., cigarette smoking, at-risk alcohol drinking, physical inactivity, and unhealthy diet) [6–11], receive fewer preventive health care services (i.e., cancer screening and vaccination) [12–16], and have chronic diseases [6, 17–20]. Furthermore, total and cardiovascular (CV) mortality increased in people with disability [21–25], and a study on elderly people aged >80 years showed that disability exerts an important influence on mortality regardless of age and multimorbidity [26]. For this reason, we hypothesized that people with disability would show higher CV disease incidence and mortality owing to higher CV risk factors.

However, most previous studies that investigated the association between disability and mortality were small studies limited to a specific population or were focused only on a specific measure of disability such as physical capability measured by handgrip strength or gait speed. To our knowledge, there is no large longitudinal general population study evaluating the association between disability and CV disease incidence and mortality, especially in Asian countries.

This study aimed to examine the association between disability and CV disease incidence and mortality in Korea longitudinally, using a national representative sample.

## Materials and methods

### Study setting

The Korean National Health Insurance Service (KNHIS) is a public health insurance that provides universal health coverage to almost all Koreans, except Medicaid beneficiaries who account for <3% of the Korean population. The KNHIS provides a National Screening Program (NSP), a biennial health screening program for all people aged >40 years and all employees regardless of age. KNHIS created the National Health Information Database (NHID), which includes data on healthcare utilization, health screening results in NSP, sociodemographic variables, and mortality for over 50 million individuals in Korea [27]. The NHID has been widely used in various epidemiological and health policy studies, and details of the database profile are described elsewhere [27, 28].

### Data sources and study population

We used the National Health Insurance Service-National Health Screening Cohort (NHIS-HEALS) database of NHID, in which 515,867 participants were included. This represents 10% of a randomly selected people from the total Korean participants aged 40–79 years who participated in the NSP at least once in 2002–2003. After excluding participants with missing data or who were misclassified, 514,679 were included in the present study.

The database included participants’ demographic data (i.e., age, sex, residential area, and income status); disability information; survey data regarding past medical history and health behaviors; and screening results including height, weight, abdominal circumference, and laboratory tests. We also obtained data on medical facility utilization and the number of deaths.

The Asan Medical Center Institutional Review Board approved this study (IRB number: 2019–0563), and the requirement for informed consent was waived because the KNHIS database was constructed after anonymization according to strict confidentiality guidelines.

### Variables

#### Independent variable

In this study, we used severity and type of disability as the independent variable, which is baseline characteristic at the time of NSP and time-invariant in our analysis model. The NHIS-HEALS dataset includes information on disability of NSP participants such as severity and type of disabilities, which were obtained from the Korean National Disability Registry. The severity of disability was divided into six grades, from 1 to 6. Grade 1 indicates extremely severe disability, while grade 6 indicates the least severe disability. In NHIS-HEALS dataset, grades 1 and 2 are categorized as “severe disability,” while grades 3–6 as “mild disability.” If participants have no disability grade, they are categorized as “normal,” which indicates that they are persons without disability. The types of disability were divided into eight categories: physical impairment, brain impairment, visual impairment, hearing impairment, linguistic impairment, mental impairment, and others (i.e., developmental disability, renal function impairment, heart function impairment, respiratory function impairment, liver function impairment, facial deformity, intestinal and urinary tract function impairment, and epilepsy). The definition of type of disability as following: physical impairment is disability related to amputation, motor disturbance, joint disability, deformity of limbs, or spinal cord injury. Brain impairment is related to brain disability caused by stroke, brain damage, or cerebral palsy. Visual impairment is related to visual loss or visual field defect, and hearing impairment is related to hearing disability or disability in sense of equilibrium. Linguistic impairment is related to mogilalia or dysphonia. Mental impairment is mental illness with limitation of daily life such as depression or schizophrenia, which is different from mental retardation that means intelligence quotient < 70. Because there were only small number of participants except physical impairment, we aggregated type of disability as following: physical, sensory (visual impairment + hearing impairment), neuropsychological (brain impairment + linguistic impairment + mental impairment + mental retardation), and others.

#### Outcome variables

CV event and mortality were outcome variables of this study. Data regarding the diagnosis of a CV event, the date of event, the cause of death, and date of death were obtained from the NHIS-HEALS database in 2002–2015. CV event or death was defined as event of or death caused by ischemic heart diseases (I20–25) or cerebrovascular disease (I60–69) during the follow-up period. If participants did not have the date of death, they were considered alive at the end of 2015. We assumed there is no right censoring other than death. Because all persons are supposed to be beneficiaries of the National Health Insurance in Korea, dropouts other than due to death are virtually impossible.

The follow-up time for CV disease was calculated as the time from the date of the NSP examination to the first diagnosis of CV disease, date of death, or the end of 2015, whichever comes first. And the follow-up time for CV mortality was calculated as the time from the date of the NSP examination to date of CV death, or the end of 2015, whichever comes first.

### Potential confounders

Potential confounders included data on health behavior, chronic diseases, and Body mass index (BMI), which are baseline characteristics at the time of NSP and time-invariant in our analysis model.

Data on health behavior were collected using a self-administered questionnaire at the time of NSP examination. In terms of smoking status, we categorized the respondents as current smokers, ex-smokers, or nonsmokers. An at-risk alcohol drinker was defined as a man <65 years who drank more than 14 drinks per week or four drinks per occasion, or a man aged ≥65 years or a woman of any age who drank more than seven drinks per week or three drinks per occasion [29].

Data on chronic diseases such as hypertension, diabetes mellitus, and dyslipidemia were collected using the same questionnaire and laboratory results of NSP. If participants responded that they took medications for hypertension, diabetes mellitus, or dyslipidemia, they were considered to have the disease. If their systolic blood pressure was ≥140 mmHg or diastolic blood pressure was ≥ 90 mmHg, they were considered to have hypertension. If their serum fasting glucose level was ≥126 mg/dL, they were considered to have diabetes mellitus. If their serum total cholesterol level was ≥240 mg/dL, they were considered to have dyslipidemia.

BMI was calculated as weight divided by height in meters squared (kg/m^2^). We used Asian-specific criteria for BMI to define normal BMI. Normal BMI is defined as 18.5 and 23 kg/m^2^, underweight as <18.5 kg/m^2^, and obese as BMI ≥ 25 kg/m^2^.

### Statistical analysis

Baseline characteristics are expressed as frequencies and percentages. We hypothesized that people with disability would show increased CV event and mortality owing to higher CV risk factors such as sex, age, health behavior, and chronic disease status compared with people without disability [2, 5]. To test this hypothesis, we conducted a survival analysis and subgroup analysis according to CV risk factors. Cox proportional hazard models were used to evaluate the association between type or severity of disability, and CV event, CV death, or all-cause death.

In the survival analyses, we built four different models. In addition to crude model, Model 1 was adjusted for sex and age. In Model 2, hypertension, diabetes mellitus, and dyslipidemia were added to Model 1. In Model 3, cigarette smoking, at-risk alcohol drinking, and BMI were added to Model 2. We calculated the hazard ratio and 95% confidence interval (CI) for each model.

We conducted subgroup analyses. First, we performed a survival analysis according to sex and age group and then according to behavioral factors such as cigarette smoking; alcohol drinking; and chronic diseases such as hypertension, diabetes mellitus, and dyslipidemia. Furthermore, we conducted a survival analysis according to aggregated type of disability. For subgroup analysis, we used the model without variable of stratification. For example, we used model 1 without sex in model 1 of subgroup analysis for sex (i.e. the model 1 in subgroup analysis for sex was adjusted only for age). Statistical analyses were performed using STATA software (version 16.1; STATA. Corp, College Station, Texas). A P-value of <0.05 was considered significant.

## Results

### Basal characteristics of participants

The mean follow-up duration was 10.8 ± 3.9 years (maximum, 13.9 years). At the end of 2015, 47,514 participants (9.2%) died. Of the deceased participants, 7,317 (1.4%) died of CV disease. In 5,572,130 person-year (PY) follow-up, the CV mortality rate was 1.313 per 1,000 PY.

Among the 514,679 participants included in the analysis, 445 (0.09%) had mild disability and 1,561 (0.30%) had severe disability. Approximately 45% of the participants were women, and the number of men with disability was higher than that of women. About 44% of total study participants were aged <50 years, while about 30% of them were aged ≥65 years. Participants with disabilities were older than those without disabilities. Over 60% of the participants had normal baseline body weight, while one-third were overweight. Two-thirds of the participants declared that they never smoked, while 23% of them answered that they smoked. The proportions of participants with disabilities who are current smokers were lesser than that of participants without disabilities. In addition, one-third of the participants revealed that they were at-risk drinkers. Half of the participants answered that they had hypertension, 12% had diabetes mellitus, and 16% had dyslipidemia (Table 1).

**Table 1:** Basic characteristics of participants.

	Total	Normal	Mild disabled	Severe disabled
	N (%)	N (%)	N (%)	N (%)
Sex (women) (%)	235,715 (45.8)	233,710 (45.9)	445 (31.3)	1,560 (36.1)
Age (years)				
<50	225,584 (43.8)	225,153 (44.2)	215 (15.1)	216 (5.0)
50–64	122,759 (23.9)	122,007 (24.0)	319 (22.5)	433 (10.0)
≥ 65	166,336 (32.3)	161,773 (31.8)	887 (62.4)	3,676 (85.0)
BMI (kg/m^2^)				
Underweight (<18.5)	12,489 (2.4)	12,267 (2.4)	75 (5.3)	147 (3.4)
Normal weight (18.5–25)	323,147 (62.8)	319,642 (62.9)	936 (66.1)	2,569 (59.4)
Overweight (≥25)	178,651 (34.7)	176,639 (34.7)	406 (28.9)	1,606 (37.2)
Cigarette smoking				
Never	329,827 (66.4)	326,137 (68.3)	944 (68.3)	2,746 (64.5)
Ex–smoker	54,006 (10.9)	53,023 (10.8)	199 (14.4)	784 (18.4)
Current smoker	112,900 (22.7)	111,930 (22.8)	240 (17.4)	730 (17.1)
At–risk alcohol drinking	71,686 (33.4)	71,221 (33.4)	140 (38.3)	325 (42.8)
Hypertension	287,211 (55.8)	283,234 (55.7)	971 (68.4)	3,016 (69.7)
Diabetes mellitus	64,008 (12.4)	62,743 (12.3)	346 (24.4)	919 (21.3)
Dyslipidemia	81,303 (15.8)	80,253 (15.8)	231 (16.3)	819 (19.0)
Cardiovascular event	3,778 (0.7)	3654 (0.7)	38 (2.7)	86 (2.0)
Cardiovascular death	7,317 (1.4)	7066 (1.4)	110 (7.7)	141 (3.3)
All-cause death	47,514 (9.2)	46,345 (9.1)	489 (34.4)	680 (15.7)

*Mild disabled: disability grades 3–6

**Severe disability: disability grades 1–2

### Cardiovascular incidence, mortality, all-cause mortality rate according to type of disability

A quarter of mild disabled and a half of severe disabled participants were physically impaired. Physical impairment was the most common type of disability in both mild and severe disabled groups in both sexes, except in women with mild disability, followed by visual and hearing impairments (Table 2).

**Table 2:** Proportion of participants by types of disability.

Type of disability	Mild disabled (N = 1,421)		Severe disabled (N = 4,325)	
	Men (N = 976)	Women (N = 445)	Men (N = 2,765)	Women (N = 1,560)
Physical impairment	249 (25.5%)	93 (20.9%)	1,365 (46.4%)	885 (56.7%)
Brain impairment	137 (14.0%)	57 (12.8%)	235 (8.5%)	105 (6.7%)
Visual impairment	103 (10.6%)	93 (21.1%)	389 (14.1%)	222 (14.2%)
Hearing impairment	159 (16.3%)	74 (16.6%)	376 (13.6%)	188 (12.1%)
Linguistic impairment	66 (6.8%)	18 (4.0%)	155 (5.6%)	50 (3.2%)
Mental retardation	69 (7.1%)	39 (8.8%)	62 (2.2%)	21 (1.4%)
Mental impairment	50 (5.1%)	26 (5.8%)	27 (1.0%)	21 (1.4%)
Others	143 (14.7%)	45 (10.1%)	156 (5.6%)	68 (4.4%)

**Fig 1:**
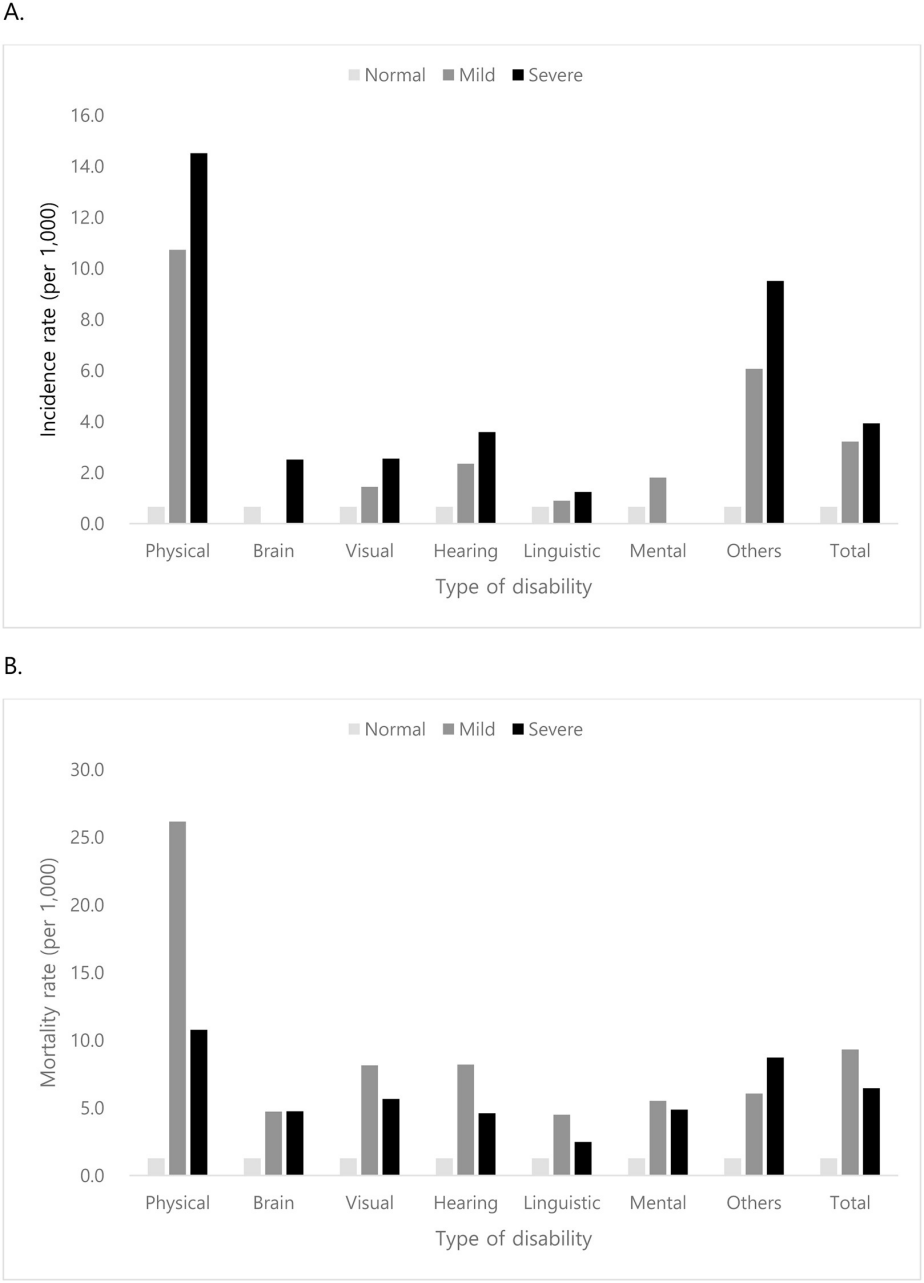
Cardiovascular disease incidence and mortality rate according to types of disability: A) Cardiovascular disease incidence. B) Cardiovascular mortality.

Among the type of disability, physically impaired participants showed the highest CV disease incidence and mortality rate in both mild and severe disabled groups (incidence rate: 10.7 per 1,000 PY for mild disabled group and 14.5 per 1,000 PY for severe disabled group; mortality rate: 26.2 per 1,000 PY for mild disabled group and 10.8 per severe disabled group). CV disease incidence and mortality were lower in patients with other types of disabilities. CV disease incidence ranged from 0 to 3.6 per 1,000 PY, while CV mortality ranged from 2.5 to 8.2 per 1,000 PY (Fig 1).

For aggregated types of disability, CV mortality, and all-cause mortality increased regardless of type of disability. The CV incidence and mortality were highest in neuropsychological disability, which contains person with previous stroke history. (Table 3).

**Table 3:** Cardiovascular disease and all-cause mortality according to type of disability.

	Disability	Number	Event	Duration (PY)	Incidence rate	aHR (95% CI)
Cardiovascular event	All	514,679	3,778	5,572,226	0.679	
	Normal	508,933	3,654	5,538,403	0.660	Ref
	Disabled	5,746	124	33,823	3.784	2.890 (2.412–3.463)
	Physical	2,592	24	10,895	2.203	1.418 (0.948–2.122)
	Sensory	1,604	21	10,905	1.926	1.322 (0.860–2.033)
	Neuropsychological	1,156	59	9,308	6.389	6.854 (5.270–8.913)
	Others	412	20	2,582	7.746	8.183 (5.207–12.857)
Cardiovascular death	All	514,679	7,317	5,572,341	1.313	
	Normal	508,933	7,066	5,538,518	1.275	Ref
	Disabled	5,746	251	33,823	7.421	2.030 (1.784–2.309)
	Physical	2,592	80	10,895	7.343	1.704 (1.362–2.132)
	Sensory	1,604	62	10,905	5.686	1.313 (1.020–1.691)
	Neuropsychological	1,156	90	9,308	9.669	4.020 (3.252–4.670)
	Others	412	19	2,582	7.359	2.846 (1.791–4.521)
All–cause death	All	514,679	47,514	5,572,341	8.527	
	Normal	508,933	46,345	5,538,518	8.368	Ref
	Disabled	5,746	1,169	33,823	34.651	1.513 (1.426–1.607)
	Physical	2,592	371	10,895	34.052	1.275 (1.149–1.414)
	Sensory	1,604	340	10,905	31.179	1.161 (1.041–1.295)
	Neuropsychological	1,156	301	9,308	32.339	2.065 (1.839–2.318)
	Others	412	157	2,582	60.805	3.608 (3.073–4.236)

Adjusted for sex, age, hypertension, diabetes mellitus, dyslipidemia, BMI, cigarette smoking, at-risk drinking

Physical: physical impairment, Sensory: visual impairment + hearing impairment, Neuropsychological: brain impairment + linguistic impairment + mental impairment + mental retardation

N/A: not applicable due to high missing rate

### Cardiovascular disease incidence and mortality according to severity of disability

CV disease incidence was significantly higher in participants with disability than in those without disability in Models 1 and 2 for both mild and severe disabled groups. However, in Model 3, CV disease incidence was three to five times higher only among participants aged 50–64 years and severe disabled participants aged <50 years. No significant difference was observed in participants aged ≥65 years. Furthermore, CV disease incidence increased in both sexes in Models 1 and 2 but not in Model 3.

Similarly, CV mortality was significantly higher in participants with disability than in those without disability in all Models. Compared with CV disease incidence, CV mortality was one and a half to six times higher in participants with disability in all age groups, except for those with severe disability aged <50 years. Moreover, CV mortality increased in both sexes in Models 1 and 2 but in only men in Model 3 (Tables 4 and 5).

**Table 4:** Association between severity of disability and cardiovascular event.

	Disability	Number	Event	Duration (PY)	Incidence rate	Crude	Model 1	Model 2	Model 3
						HR (95% CI)	aHR (95% CI)	aHR (95% CI)	aHR (95% CI)
Total	All	514,679	3,778	5,572,015	0.678				
	Normal	508,933	3,654	5,538,329	0.660	Ref	Ref	Ref	Ref
	Mild	1,421	38	11,821	3.214	6.297(4.574–8.670)	2.748(1.994–3.788)	2.758(2.001–3.800)	1.394(0.578–3.360)
	Severe	4,325	86	21,864	3.933	7.945(6.413–9.843)	2.822(2.273–3.502)	2.868(2.311–3.560)	1.548(0.927–2.585)
Sex									
Men	All	278,694	2,496	3,023,064	0.826				
	Normal	275,223	2,407	3,000,020	0.802	Ref	Ref	Ref	Ref
	Mild	976	29	8,052	3.602	5.908 (4.095–8.523)	2.507 (1.736–3.620)	2.505 (1.734–3.617)	1.469 (0.609–3.542)
	Severe	2,765	60	14,992	4.002	6.700 (5.183–8.661)	2.412 (1.862–3.124)	2.431 (1.876–3.148)	1.456 (0.840–2.524)
Women	All	235,715	1,282	2,548,951	0.503				
	Normal	233,710	1,247	2,538,309	0.491	Ref	Ref	Ref	Ref
	Mild	445	9	3,770	2.388	6.226 (3.231–11.996)	3.629 (1.882–6.995)	3.620 (1.878–6.979)	N/A
	Severe	1,560	26	6,873	3.783	10.466 (7.093–15.442)	4.279 (2.894–6.326)	4.408 (2.981–6.516)	2.359 (0.577–9.637)
Age									
<50	All	225,584	731	2,891,196	0.253				
	Normal	225,153	726	2,885,839	0.252	Ref	Ref	Ref	Ref
	Mild	215	2	2,638	0.758	3.826(0.955–15.329)	3.327(0.830–13.333)	3.267(0.815–13.097)	5.481(0.769–39.085)
	Severe	216	3	2,718	1.104	4.359(1.402–13.547)	3.655(1.176–11.364)	3.567(1.147–11.091)	5.570(1.386–22.376)
50–64	All	122,759	1,149	1,490,664	0.771				
	Normal	122,007	1,117	1,482,506	0.753	Ref	Ref	Ref	Ref
	Mild	319	14	3,310	4.229	7.822(4.616–13.254)	6.033(3.557–10.232)	5.787(3.410–9.822)	4.590(1.469–14.343)
	Severe	433	18	4,848	3.713	5.978(3.752–9.525)	4.498(2.819–7.176)	4.541(2.846–7.245)	3.349(1.248–8.991)
≥ 65	All	166,336	1,898	1,190,155	1.595				
	Normal	161,773	1,811	1,169,984	1.548	Ref	Ref	Ref	Ref
	Mild	887	22	5,873	3.745	2.496(1.639–3.800)	2.232(1.465–3.401)	2.232(1.465–3.401)	0.411(0.058–2.926)
	Severe	3,676	65	14,298	4.546	3.198(2.496–4.096)	2.916(2.273–3.741)	2.921(2.277–3.748)	1.396(0.720–2.705)
Health behavior									
Current smoker	All	112,900	810	1,287,568	0.628				
	Normal	111,930	785	1,280,710	0.613	Ref	Ref	Ref	Ref
	Mild	240	4	2,209	1.811	4.122(1.542–11.015)	1.904(0.712–5.094)	1.905(0.712–5.098)	1.776(0.442–7.139)
	Severe	730	20	4,648	4.303	9.818(6.293–15.317)	3.236(2.063–5.077)	3.281(2.091–5.148)	2.675(1.372–5.213)
At-risk drinking	All	71,686	483	792,504	0.609				
	Normal	71,221	473	788,162	0.600	Ref	Ref	Ref	Ref
	Mild	140	4	1,367	2.927	6.207(2.319–16.615)	2.893(1.079–7.760)	2.867(1.069–7.688)	2.956(1.102–7.927)
	Severe	325	6	2,976	2.016	4.250(1.899–9.514)	1.284(0.571–2.888)	1.312(0.583–2.952)	1.384(0.615–3.115)
Chronic disease									
Hypertension	All	287,221	2,631	2,953,790	0.891				
	Normal	283,234	2,541	2,931,174	0.867	Ref	Ref	Ref	Ref
	Mild	971	27	7,730	3.493	5.107(3.495–7.464)	2.711(1.853–3.965)	2.682(1.833–3.923)	0.749(0.187–3.001)
	Severe	3,016	63	14,886	4.232	6.352(4.945–8.159)	2.762(2.146–3.555)	2.786(2.165–3.586)	1.313(0.701–2.457)
Diabetes mellitus	All	64,008	658	581,099	1.132				
	Normal	62,743	628	574,878	1.092	Ref	Ref	Ref	Ref
	Mild	346	12	2,485	4.828	5.103(2.882–9.037)	3.586(2.023–6.355)	3.592(2.207–6.366)	1.226(0.172–8.751)
	Severe	919	18	3,736	4.818	5.688(3.556–9.098)	2.907(1.812–4.665)	2.936(1.829–4.712)	1.760(0.559–5.539)
Dyslipidemia	All	81,303	643	806,647	0.797				
	Normal	80,253	623	801,370	0.777	Ref	Ref	Ref	Ref
	Mild	231	8	1,851	4.322	7.118(3.542–14.306)	3.652(1.812–7.358)	3.589(1.781–7.232)	2.072(0.289–14.849)
	Severe	819	12	3,427	3.502	5.825(3.286–10.323)	2.765(1.556–4.914)	2.827(1.591–5.026)	0.852(0.119–6.124)

Model 1: sex and age

Model 2: Model 1 + hypertension, diabetes mellitus, and dyslipidemia

Model 3: Model 2 + body mass index, cigarette smoking, and at-risk alcohol drinking

For subgroup analysis, we used the model without variable of stratification. For example, we used model 1 without sex in model 1 of subgroup analysis for sex

N/A: not applicable due to high missing rate, CI: confidence interval, PY: person-year, HR: hazard ratio

**Table 5:** Association between severity of disability and cardiovascular death.

Age	Disability	Number	Death	Duration (PY)	Mortality rate	Crude	Model 1	Model 2	Model 3
						HR (95% CI)	aHR (95% CI)	aHR (95% CI)	aHR (95% CI)
Total	All	514,679	7,317	5,572,130	1.313				
	Normal	508,984	7,066	5,538,444	1.275	Ref	Ref	Ref	Ref
	Mild	1,421	110	11,821	9.305	7.543 (6.248–9.106)	2.908 (2.407–3.513)	2.905 (2.403–3.512)	3.182 (2.217–4.567)
	Severe	4,325	141	21,864	6.449	5.426 (4.592–6.411)	1.586 (1.341–1.876)	1.613 (1.364–1.907)	1.729 (1.285–2.327)
Sex									
Men	All	278,962	4,401	3,023,155	1.456				
	Normal	275,223	4,213	3,000,112	1.404	Ref	Ref	Ref	Ref
	Mild	976	84	8,052	10.432	7.618 (6.138–9.456)	3.011 (2.424–3.740)	3.032 (2.441–3.765)	3.402 (2.369–4.885)
	Severe	2,765	106	14,992	7.071	5.316 (4.386–6.447)	1.688 (1.390–2.049)	1.717 (1.414–2.084)	1.718 (1.258–2.345)
Women	All	235,715	2,914	2,548,975	1.143				
	Normal	233,710	2,853	2,538,332	1.124	Ref	Ref	Ref	Ref
	Mild	445	26	3,770	6.897	6.384 (4.339–9.393)	2.927 (1.989–4.307)	2.810 (1.895–4.167)	N/A
	Severe	1,560	35	6,873	5.092	4.987 (3.572–6.961)	1.444 (1.034–2.017)	1.451 (1.039–2.027)	1.790 (0.661–4.849)
Age									
<50	All	225,584	604	2,891,255	0.208				
	Normal	225,153	597	2,885,899	0.206	Ref	Ref	Ref	Ref
	Mild	215	6	2,638	1.022	11.092 (4.963–24.788)	9.064 (4.053–20.269)	8.166 (3.648–18.278)	6.250 (1.553–25.152)
	Severe	216	1	2,718	0.368	1.788 (0.251–12.715)	1.470 (0.207–10.458)	1.404 (0.197–9.988)	2.539 (0.356–18.090)
50–64	All	122,759	2,022	1,490,703	1.356				
	Normal	122,007	1,977	1,482,544	1.334	Ref	Ref	Ref	Ref
	Mild	319	22	3,310	6.646	5.089 (3.343–7.747)	3.407 (2.237–5.190)	2.946 (1.915–4.534)	3.459 (1.641–7.289)
	Severe	433	23	4,848	4.744	3.594 (2.383–5.422)	2.269 (1.503–3.426)	2.263 (1.499–3.416)	2.550 (1.320–4.925)
≥65	All	166,336	4,691	1,190,170	3.941				
	Normal	161,773	4,492	1,169,999	1.839	Ref	Ref	Ref	Ref
	Mild	887	82	5,873	13.962	3.670 (2.950–4.566)	2.688 (2.159–3.346)	2.720 (2.185–3.386)	2.709 (1.756–4.178)
	Severe	3,676	117	14,298	6.827	2.277 (1.895–2.735)	1.489 (1.238–1.791)	1.497 (1.245–1.801)	1.483 (1.057–2.081)
Health behavior									
Current smoker	All	112,900	1,997	1,287,632	1.549				
	Normal	111,930	1,947	1,280,774	1.519	Ref	Ref	Ref	Ref
	Mild	240	23	2,209	10.411	6.935 (4.597–10.462)	2.931 (1.941–4.425)	3.017 (1.998–4.556)	2.985 (1.687–5.281)
	Severe	730	27	4,648	5.809	3.965 (2.711–5.797)	1.121 (0.765–1.642)	1.162 (0.793–1.702)	1.270 (0.761–2.120)
At-risk drinking	All	71,686	1,162	792,527	1.466				
	Normal	71,221	1,128	788,186	1.431	Ref	Ref	Ref	Ref
	Mild	140	11	1,367	8.049	5.719 (3.158–10.257)	2.430 (1.340–4.405)	2.473 (1.364–4.484)	2.610 (1.439–4.732)
	Severe	325	23	2,976	7.730	5.546 (3.670–8.381)	1.592 (1.050–2.413)	1.653 (1.091–2.506)	1.526 (0.988–2.358)
Chronic disease									
Hypertension	All	287,221	5,668	2,953,874	1.919				
	Normal	283,234	5,476	2,931,258	1.868	Ref	Ref	Ref	Ref
	Mild	971	83	7,730	10.737	5.974 (4.810–7.421)	2.858 (2.300–3.553)	2.825 (2.274–3.516)	3.169 (2.098–4.787)
	Severe	3,016	109	14,886	7.322	4.216 (3.487–5.096)	1.593 (1.317–1.928)	1.601 (1.323–1.938)	1.779 (1.282–2.469)
Diabetes mellitus	All	64,008	1,565	581,109	2.693				
	Normal	62,743	1,504	574,888	2.616	Ref	Ref	Ref	Ref
	Mild	346	30	2,485	12.070	4.812 (3.352–6.908)	3.390 (2.361–4.868)	3.280 (2.271–4.739)	2.951 (1.319–6.602)
	Severe	919	31	3,736	8.298	3.071 (2.878–3.277)	1.725 (1.206–2.466)	1.697 (1.187–2.427)	2.450 (1.375–4.366)
Dyslipidemia	All	81,303	1,262	806,647	1.565				
	Normal	80,253	1,228	801,370	1.532	Ref	Ref	Ref	Ref
	Mild	231	16	1,851	8.644	5.819 (3.553–9.529)	2.640 (1.610–4.329)	2.631 (1.604–4.314)	3.146 (1.171–8.448)
	Severe	819	18	3,427	5.253	3.745 (2.351–5.966)	1.541 (0.966–2.458)	1.583 (0.992–2.526)	0.984 (0.314–3.081)

Model 1: sex and age

Model 2: Model 1 + hypertension, diabetes mellitus, and dyslipidemia

Model 3: Model 2 + BMI, cigarette smoking, and at-risk alcohol drinking

For subgroup analysis, we used the model without variable of stratification. For example, we used model 1 without sex in model 1 of subgroup analysis for sex

N/A: not applicable due to high missing rate, CI: confidence interval, PY: person-year, HR: hazard ratio

### Subgroup analysis

For current smokers, CV disease incidence was significantly increased in the severe disabled group in all Models, whereas CV mortality was increased only in the mild disabled group in all Models. At-risk drinkers with mild disability were more likely to have CV event in all Models. Similarly, at risk-drinkers with disability showed higher CV mortality, except for participants with severe disability, in Model 3.

Participants with hypertension or diabetes showed increased CV disease incidence when they had a disability in Models 1 and 2 but not in Model 3, while their mortality was significantly higher in all Models than that of participants without disability. Similarly, CV disease incidence was higher in participants with dyslipidemia in both mild and severe disability groups in Models 1 and 2, while only those with mild disability had higher CV incidence in Model 3. For CV mortality, participants with mild disability showed higher mortality in all Models when they had dyslipidemia.

## Discussion

To our knowledge, this is the first large general population study to evaluate the association of CV disease incidence and mortality with disability in the Asian population, using a nationwide representative claim database. Overall, CV disease incidence, CV mortality, and all-cause mortality increased in participants with disability regardless of type or severity of disability after full adjustment of CV risk factors. This finding showed that CV mortality increase in people with disability is not fully explained by their higher CV risk factors. Although CV mortality increased in all age groups, the increase was more evident in young participants. Although CV disease incidence and mortality were not significantly different in the subgroup analysis of health behavior in the fully adjusted model, CV mortality increase was observed in the subgroup analysis of participants with chronic diseases.

Several previous studies showed that mortality increased in people with physical frailty or disability. A systematic review of 19 studies evaluating the association of handgrip strength and gait speed with CV mortality also showed that decreased physical performance was associated with increased CV mortality [23]. In the study, they found that the association was independent of anthropometric features (i.e., BMI and height) and traditional CV risk factors (i.e., smoking and dyslipidemia). However, the studies included in the review used a limited definition of physical disability measured by handgrip strength or gait speed, and no large-scale long-term study was conducted in the Asian population.

A previous study on older adults aged ≥80 years showed that disability was more predictive of all-cause mortality than multimorbidity [26]. In the study, people with disability had 2.36 times more risk of mortality than those without disability or comorbidity, while people with multimorbidity had 1.66 times more risk of mortality. This finding indicated that disability itself contributed to mortality in the study population regardless of comorbidities in people with disability. Similarly, we found that CV disease incidence and mortality increased after controlling chronic diseases (i.e., Model 2), and subgroup analyses of chronic diseases showed the same results. Based on these results, we assumed that disability is among the risk factors for CV regardless of chronic diseases, which are known traditional risk factors for CV.

The current study showed that CV mortality increased only in men in the fully adjusted model (i.e., Model 3). This finding suggests that there is discrepancy between sexes in terms of the effect of disability on CV mortality. A similar finding was reported in a Finnish study of 4,501 men and women aged ≥45 years, which showed that disability was predictive of mortality only in men not in women with coronary heart disease (CHD) [30]. In the study, the incidence of coronary heart disease-related death increased 1.3–1.5 times in men and women, except in women with a history of CHD, which indicates that risk factors for CV disease differ between men and women. This discrepancy is possibly explained by differences in sex hormone, which influences CV risk and physical performance in different ways in both sexes [31–33].

We found that the effect of disability on CV disease incidence and mortality was not necessarily greater in older participants. CV mortality was six times higher in participants aged <50 years with mild disability, while it was less than four times in those aged ≥50 years. Although this study showed that disability was a risk factor for CV disease independent of conventional risk factors, our finding suggests that the effect of disability on CV disease is diluted when participants have more conventional CV risk factors, which are far more prevalent in older adults.

There are several limitations in this study. There is a limitation in the database, which prevented the adjustment for well-known CV risk factors such as education and dietary intake. Moreover, the database did not include data on the severity of CV disease; therefore, the models cannot be adjusted for severity of the disease. Furthermore, we collected data on health behaviors using a questionnaire; thus, it is possible that there is recall bias in those data. We did not include physical activity as a covariate due to the large amount of missing information in the database, which is an important health behavior for CV disease prevention. Additionally, because NHID is a medical claims database, it is possible that the diagnoses of CV disease and cause of death were misclassified. However, all claims in this dataset were audited by the Korean Health Insurance Review and Assessment before payment, making the misclassification of diagnosis improbable.

In conclusion, people with disability showed higher CV disease incidence and mortality than those without disability, regardless of the type of disability or risk factors for CV disease.
